# Distinct Signaling Functions of Rap1 Isoforms in NO Release From Endothelium

**DOI:** 10.3389/fcell.2021.687598

**Published:** 2021-06-17

**Authors:** Ramoji Kosuru, Bandana Singh, Sribalaji Lakshmikanthan, Yoshinori Nishijima, Jeannette Vasquez-Vivar, David X. Zhang, Magdalena Chrzanowska

**Affiliations:** ^1^Blood Research Institute, Versiti, Milwaukee, WI, United States; ^2^Department of Medicine, Medical College of Wisconsin, Milwaukee, WI, United States; ^3^Department of Biophysics and Redox Biology Program, Medical College of Wisconsin, Milwaukee, WI, United States; ^4^Cardiovascular Center, Medical College of Wisconsin, Milwaukee, WI, United States; ^5^Department of Pharmacology and Toxicology, Medical College of Wisconsin, Milwaukee, WI, United States

**Keywords:** endothelial (dys)function, vasodilation, nitric oxide signaling, small GTP protein, Rap1A, Rap1B

## Abstract

Small GTPase Rap1 plays a prominent role in endothelial cell (EC) homeostasis by promoting NO release. Endothelial deletion of the two highly homologous Rap1 isoforms, Rap1A and Rap1B, leads to endothelial dysfunction *ex vivo* and hypertension *in vivo*. Mechanistically, we showed that Rap1B promotes NO release in response to shear flow by promoting mechanosensing complex formation involving VEGFR2 and Akt activation. However, the specific contribution of the Rap1A isoform to NO release and the underlying molecular mechanisms through which the two Rap1 isoforms control endothelial function are unknown. Here, we demonstrate that endothelial dysfunction resulting from knockout of both Rap1A and Rap1B isoforms is ameliorated by exogenous L-Arg administration to rescue NO-dependent vasorelaxation and blood pressure. We confirmed that Rap1B is rapidly activated in response to agonists that trigger eNOS activation, and its deletion in ECs attenuates eNOS activation, as detected by decreased Ser1177 phosphorylation. Somewhat surprising was the finding that EC deletion of Rap1A does not lead to impaired agonist-induced vasorelaxation *ex vivo*. Mechanistically, the deletion of Rap1A led to elevated eNOS phosphorylation both at the inhibitory, T495, and the activating Ser1177 residues. These findings indicate that the two Rap1 isoforms act *via* distinct signaling pathways: while Rap1B directly positively regulates eNOS activation, Rap1A prevents negative regulation of eNOS. Notably, the combined deficiency of Rap1A and Rap1B has a severe effect on eNOS activity and NO release with an *in vivo* impact on endothelial function and vascular homeostasis.

## Introduction

Located at the blood interface, the endothelium controls vessel and organ function by releasing bioactive substances. In response to physical and chemical cues from the environment: laminar shear stress of flowing blood and agonists: bradykinin, acetylcholine, and ATP, endothelial-derived factors modulate vascular smooth muscle tone, maintain non-adhesive lumen surface, and control inflammatory responses in the vascular wall, thereby controlling blood flow. Among EC-derived factors, endothelial nitric oxide synthase NOS (eNOS, NOS3)-produced NO release is fundamental to vascular homeostasis as disruption of NO production leads to endothelial dysfunction and underlies cardiovascular disease (Widlansky et al., 2003; Vanhoutte et al., 2009). eNOS activity is regulated by Ca^2+^ and Ca^2+^-regulatory protein, calmodulin, binding of regulatory cofactors, and post-translational modifications, including palmitoylation and phosphorylation (Michel and Feron, 1997). Phosphorylation of Ser1177 and Ser633 sensitizes eNOS to Ca^2+^ and stimulates NO production, whereas Thr495 phosphorylation is inhibitory (Förstermann and Sessa, 2012). We have recently identified small GTPase Rap1 (Ras Association Proximate) (Lakshmikanthan et al., 2015) as a novel factor controlling eNOS-dependent NO release.

Two closely related Rap1 isoforms, Rap1A and Rap1B, are key regulators of vascular homeostasis (Chrzanowska-Wodnicka, 2017). Many studies, in particular in isolated ECs, point to the role of Rap1 in the modulation of cell–matrix and cell–cell adhesion (Boettner and Van Aelst, 2009). The presence of either isoform is required for *de novo* cell–cell junction formation *in vitro* (Lakshmikanthan et al., 2018) and normal vasculogenesis *in vivo* (Chrzanowska-Wodnicka et al., 2015). Interestingly, EC deletion of both Rap1 isoforms after birth and establishment of vascular barriers does not increase vascular permeability in most vascular beds (Lakshmikanthan et al., 2018). Instead, the most profound defect in total Rap1 (Rap1A + Rap1B), EC-specific KO (Rap1^iΔEC^) mice is endothelial dysfunction due to a decreased NO bioavailability defect, evidenced by impaired vasorelaxation defect *ex vivo* and hypertension *in vivo* (Lakshmikanthan et al., 2015). Endothelial deletion of Rap1B, the predominant EC Rap1 isoform, leads to a partial vasorelaxation defect (Lakshmikanthan et al., 2014) and impaired NO release in response to shear stress (Lakshmikanthan et al., 2015). Mechanistically, in response to shear flow, Rap1B acts as a mechanosensor promoting the formation of the endothelial junctional mechanosensing complex, VEGFR2 transactivation, and signaling to NO release involving Akt recruitment and Ser1177 phosphorylation of eNOS (Lakshmikanthan et al., 2015). However, the mechanism through which Rap1 controls agonist-induced NO release or the individual contribution of each of the Rap1 isoforms to the control of NO release is unknown.

Herein, taking advantage of novel EC-specific Rap1A (Rap1A^iΔEC^) and Rap1 (1A + 1B, Rap1^iΔEC^) knockout mice, we investigated the isoforms’ contribution to NO release *ex vivo*. Specifically, we examined the involvement of each Rap1 isoform on eNOS phosphorylation at key regulatory sites. Our results confirm the cooperation between the two isoforms in promoting NO release, while they also reveal dramatically distinct functions of the two isoforms in signaling to eNOS and NO release. Physiologically significant, our results bring new information on Rap1 endothelial biology.

## Materials and Methods

### Animals, L-Arginine Treatment, and Blood Pressure Measurement

All mouse procedures were performed according to the Medical College of Wisconsin Institutional Animal Use and Care Committee. Generation of endothelial cell (EC)-specific total Rap1 KO mice [Rap1A + Rap1B knockout, Cadh5(PAC)-CreERT2^+/0^; Rap1A^f/f^ Rap1B^f/f^; Rap1^i^**Δ**^EC^] and EC-specific Rap1A-knockout mice [Cadh5(PAC)-CreERT2^+/0^; Rap1A^f/f^ Rap1B^+/+^; Rap1A^i^**Δ**^EC^] on C57Bl/6J background was previously described (Lakshmikanthan et al., 2015, 2018). Cadh5-Cre-negative mice, or mice injected with carrier oil only, were used as controls. For L-arginine treatment, drinking water was supplemented with L-arginine (6 g/kg/day) (Chen et al., 2003) at weaning until the analyses were completed; control mice received plain water. Systolic blood pressure measurements were performed thrice-weekly in conscious 8- to 12-week-old male and female animals using IITC Life Science Blood Pressure System, as described previously (Kriska et al., 2012; Lakshmikanthan et al., 2015).

### Aortic Ring Wire Myography

Endothelium-dependent relaxation of aortic rings in response to acetylcholine was measured as previously described (Mendoza et al., 2010; Lakshmikanthan et al., 2015). Briefly, the thoracic aortae from control and Rap1^i^**Δ**^EC^ mice were dissected and placed in Krebs physiological saline aerated with 21% O_2_/5% CO_2_. Artery segments were mounted on parallel pins in conventional myographs and maintained at 37°C in Krebs buffer for 60 min. The vessels were then preconstricted with 10 nM phenylephrine or 10 nM thromboxane mimetic U-46619. After constriction, relaxation responses to the increasing concentration of acetylcholine were recorded.

### Cell Culture, Transfection, and Treatments

Primary human coronary arteriole endothelial cells (hCAECs) (Lonza #CC-2585) and human pulmonary aorta ECs were cultured with standard methods for no longer than six passages and were used for all *in vitro* experiments. For 30–40% confluent EC, monolayers were transfected with 50 nM Rap1A or Rap1B siGENOME siRNA pool, or with non-targeting siRNA pool (Dharmacon) for 6 h and cultured for an additional 30 h in complete EBM culture medium (Lonza#CC-3156). Prior to biochemical analysis, cells were serum-starved for 4–6 h. To induce eNOS phosphorylation, cells were treated with 500 μM ATP. Ach (10 μM, 60 min)- or bradykinin (1 μM, 60 min)-induced NO release in hPAECs was estimated from the reduction of nitrite to NO and quantified by NO-Analyzer-chemiluminescence using authentic standards as previously described (Cornelius et al., 2009).

### Rap1 Activity Assay and Western Blotting

Active, GTP-bound fraction of Rap1 in lysates of ECs subjected to 1 μM bradykinin was measured using RalGDS-GST pull-down method, as previously described (Franke et al., 2000; Lakshmikanthan et al., 2015). Agonist-induced eNOS phosphorylation in ECs was assessed by Western blotting of Tris-glycine 4–12% gradient gel-resolved cell lysates, blotted onto nitrocellulose membranes as previously described (Lakshmikanthan et al., 2015; Singh et al., 2021). The following antibodies were used for Western Blot analysis: antibodies against phospho-eNOS (Ser-1177) (BD Biosciences #612392), phospho-eNOS (Thr495) (Cell Signaling Technologies #9574), total eNOS (Cell Signaling Technologies #32027), Rap1A/Rap1B (Cell Signaling Technologies #2399), and β-actin (Santa Cruz Biotechnology #sc-47778). Rap1 rabbit monoclonal antibody (clone 26B4, Cell Signaling Technologies #2399) was used for GTP-Rap1 pull-down assay. Horseradish peroxidase-conjugated secondary antibodies followed by chemiluminescence detection using Amersham Imager 600 and analysis software (GE Healthcare) were used for densitometry. Blanked values (following subtraction of the corresponding empty well lane value), normalized within each experiment, were used to calculate fold change in phosphorylation between Rap1-deficient and control conditions.

### Statistical Analysis

GraphPad Prism version 5 (GraphPad Software) was used. Statistical significance of group differences was determined using two-tailed unpaired Student’s *t*-test with Welch’s correction; ^∗^*p* < 0.05; ^∗∗^*p* < 0.01; ^∗∗∗^*p* < 0.001.

## Results

### Endothelial Dysfunction Resulting From Rap1 Deficiency Is Ameliorated by Exogenous L-Arginine Supplementation

**FIGURE 1:**
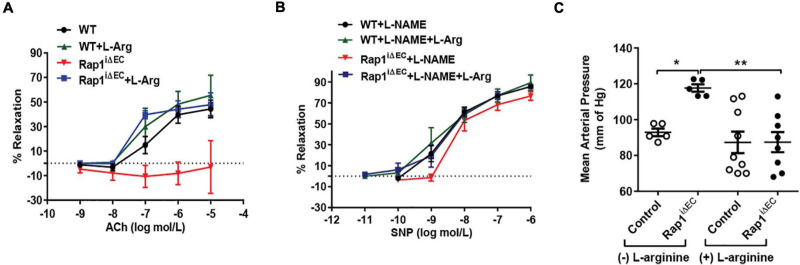
Endothelial dysfunction in Rap1i^ΔEC^ mice is attenuated by L-arginine (L-Arg) supplementation. **(A,B)** Corrected vasorelaxation. Concentration dependent vasorelaxation of U466169-preconstricted aortae from control (WT) and Rap1^i^**^Δ^**^EC^ mice treated with or without L-arginine in response to ACh **(A)**, sodium nitroprusside (SNP) **(B)**. SNP response was measured in presence of L-NAME, an NO blocker. Values are means ± SEM (*n* = 6). **(C)** L-arginine treatment normalizes blood pressure in Rap1^iΔEC^ mice. Systolic blood pressure was measured in control (WT) and Rap1^iΔEC^ mice with or without L-arginine supplementation in drinking water. Values are means + SEM (*n* = 5–9).* indicates *p* < 0.05 vs. control without L-arginine; ** indicates *p* < 0.01 vs. Rap1^iΔEC^ without L-arginine. One-way ANOVA Tukey’s multiple comparison test was applied to compare statistical significance.

Endothelial Rap1 deficiency of both Rap1 isoforms, Rap1A and Rap1B, significantly impairs endothelial NO production *in vitro* and NO-dependent vasorelaxation, leading to endothelial dysfunction and hypertension *in vivo* (Lakshmikanthan et al., 2015). This effect is concentration-dependent, with partial decrease in single allele, Rap1B-KO mice (Lakshmikanthan et al., 2014). Exogenous administration of NO synthesis precursor, L-arginine as substrate to the ECs increases NO availability and leads to increased vascular relaxation *in vivo* (Palmer et al., 1988; Böger et al., 1998). To test if NO is the main effector of Rap1 in endothelium *in vivo* responsible for endothelial dysfunction in Rap1^i^**Δ**^EC^ mice, we exogenously administered L-arginine to EC-specific Rap1 knockout (Rap1^i^**Δ**^EC^) mice to compensate for decreased eNOS activity and measured endothelium-dependent responses. Depressed NO-dependent vasorelaxation of aortic segments from Rap1^i^**Δ**^EC^ mice (Lakshmikanthan et al., 2015) was restored upon L-arginine treatment to the level similar to control mice (Figure 1A). To determine vascular specificity of the L-arginine effect, we blocked NO release with L-nitro-arginine methyl ester (L-NAME) and measured vasorelaxation in the presence of sodium nitroprusside (SNP, EC-independent vasorelaxation). We found that the extent of SNP-induced vasorelaxation of aortic segments was unchanged in Rap1^i^**Δ**^EC^ aortae compared to controls (Figure 1B). Likewise, blood pressure, elevated in Rap1^i^**Δ**^EC^ mice (Lakshmikanthan et al., 2015), was lowered to normal levels with L-arginine treatment (Figure 1C). These findings demonstrate that compensation for depressed NO production is sufficient to overcome Rap1 deficiency-induced endothelial dysfunction *in vivo* and suggests that NO is the major Rap1 effector in maintaining vessel homeostasis.

### Rap1B Acts in a GPCR-Induced Signaling Pathway to eNOS Activation

**FIGURE 2:**
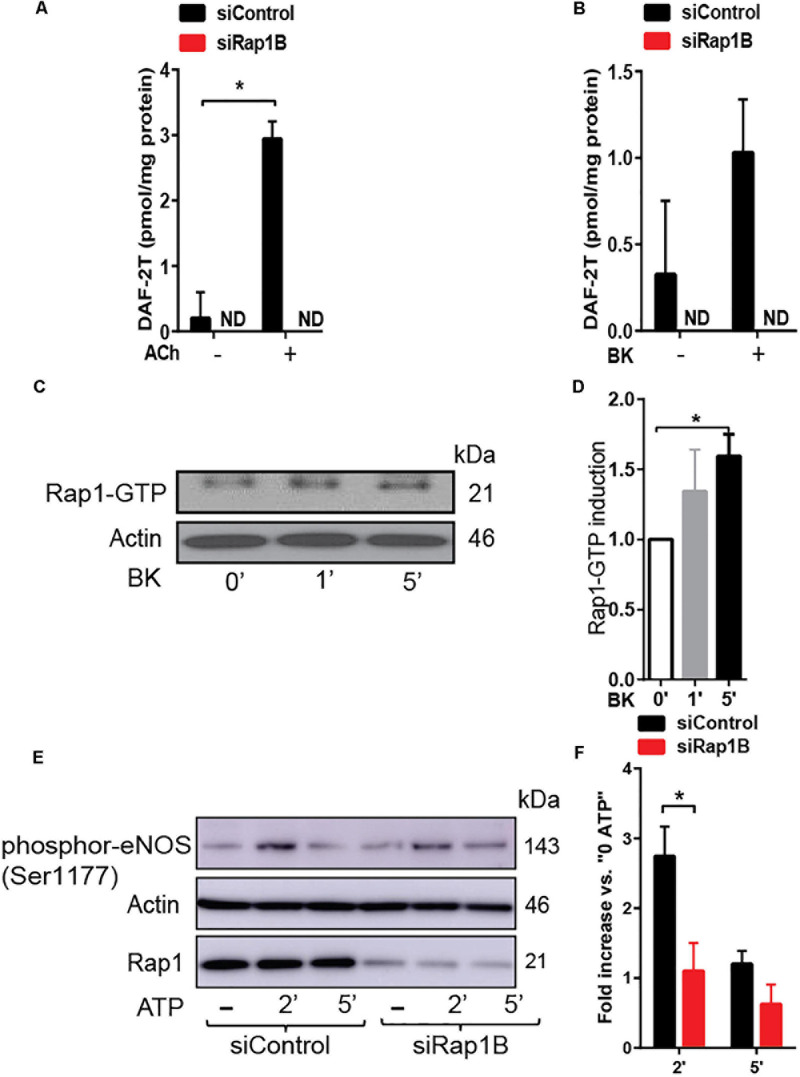
**(A,B)** RaplB deletion inhibits agonist-induced NO release. NO release measured in HPAECs preincubated with 2 ml DAF-DA (5 μM) + L-arginine 10 μM with or without ACh **(A)** and bradykinin (BK) **(B)** for 30 min at 37°C. Mean values of concentrations calculated using authentic standards are shown (*n* = 3). ND: not dected. * indicates *p* < 0.05 vs. siControl unstimulated. One-way ANOVA Tukey’s multiple comparison test was applied to compare statistical significance. **(C,D)** Rap1 is activated by GPCR agonist that induced nitric oxide (NO). Bradykinin (BK)-induced, GTP-bound Rap1 using Rap1-GTP pulldown assay. Representative immunoblot **(C)** and quantification **(D)** of Rap1 bound to GST-RalGDS beads following 1 and 5 min treatment with BK. Values are means ± SEM (*n* = 3). * indicates *p* < 0.05 vs. 0’ BK. Unpaired *t*-test was applied to compare statistical significance. **(E,F)** Rap1B deletion attenuates agonist-induced Ser-1177 eNOS phosphorylation. Representative immunoblot **(E)** and quantification **(F)** of fold increase of phosphor-eNOS (Ser1177) vs.0 ATP in siControl and siRap1B HCAECs (*n* = 4). * indicates *p* < 0.05 vs. siControl 2’ ATP. One-way ANOVA Tukey’s multiple comparison test was applied to compare statistical significance.

Decreased vasorelaxation response to ACh of aortic segments from Rap1B^iΔEC^ mice suggested that Rap1B is required for agonist-induced NO release. To test this hypothesis directly, we examined the effect of Rap1B deletion on agonist-induced NO release from ECs. In the absence of stimulation, the release of NO was negligible in siControl ECs and not detected in siRap1B ECs (Figure 2A). Treatment of ECs with acetylcholine or bradykinin induced significant release of NO (Figure 2A). Interestingly, knockdown of Rap1B inhibited bradykinin-induced NO release from ECs (Figure 2B). As this finding supported the role of Rap1B in a signaling pathway from GPCR-linked agonist receptors to eNOS activation, we examined the ability of bradykinin to induce Rap1 activation in hCAECs (Figure 2C). Consistently with previous results (Lakshmikanthan et al., 2015), we found a considerable level of active Rap1 in quiescent ECs (Figure 2D). Nonetheless, bradykinin induced a rapid and significant increase in active Rap1 fraction (Figure 2D), consistent with Rap1 acting downstream from GPCR. Next, to determine the involvement of Rap1B in eNOS activation, we examined the effect of siRap1B knockdown on agonist (ATP)-induced eNOS/Ser1177 phosphorylation (Figure 2E). Agonist induced rapid and transient eNOS-Ser1177 phosphorylation in siControl ECs (Figure 2F). Levels of eNOS-Ser1177 were significantly decreased in siRap1B ECs (Figure 2F), consistent with the observed decreased NO release from siRap1B ECs (Figures 2A,B). Altogether, these data support a model in which GPCR-induced Rap1B activity promotes agonist-induced eNOS activation (Figure 4).

**FIGURE 3:**
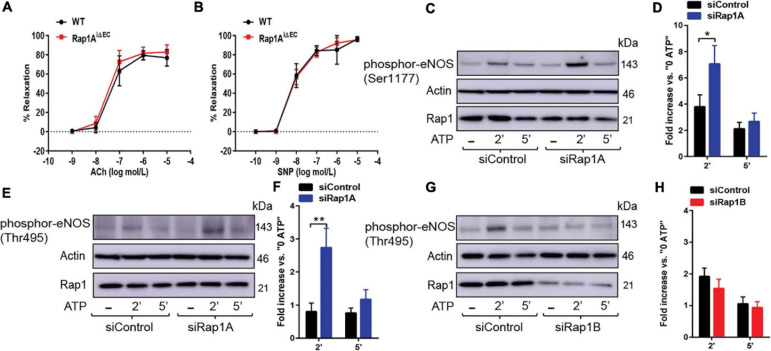
**(A,B)** Vasorelaxation is not impaired upon EC-deletion of Rap1A isoform alone. Concentration dependent vasorelaxation of phenylephrine-preconstricted aortae from control (WT) and Rap1A^iΔEC^ mice treated with ACh **(A)**, and SNP **(B)**. Values are means ± SEM (*n* = 6). **(C,D)** eNOS Ser1177 phosphorylation is increased in siRap1A ECs. Representative Immunoblot **(C)** and quantification **(D)** of fold increase of Ser1177 vs.0 ATP in siControl and siRap1A HCAECs (*n* = 6). * indicates *p* < 0.05 vs. siControl 2’ ATP. One-way ANOVA Tukey’s multiple comparison test was applied to compare statistical significance. **(E,F)** siRap1A knockdown leads to elevated phosphor-eNOS (Thr-495). Representative Immunoblot **(E)** and quantification **(F)** of fold increase of phosphor-eNOS (Thr495) vs.0 ATP in siControl and siRap1A HCAECs (*n* = 5). **(G,H)** siRap1B leads to decreased eNOS Thr-495 phosphorylation. Representative Immunoblot **(G)** and quantification **(H)** of fold increase of phosphor-eNOS T(hr495) vs.0 ATP in siControl and siRap1B HCAECs (*n* = 5). ** indicates *p* < 0.01.vs. siControl 2’ ATP. One-way ANOVA Tukey’s multiple comparison test was applied to compare statistical significance.

**FIGURE 4:**
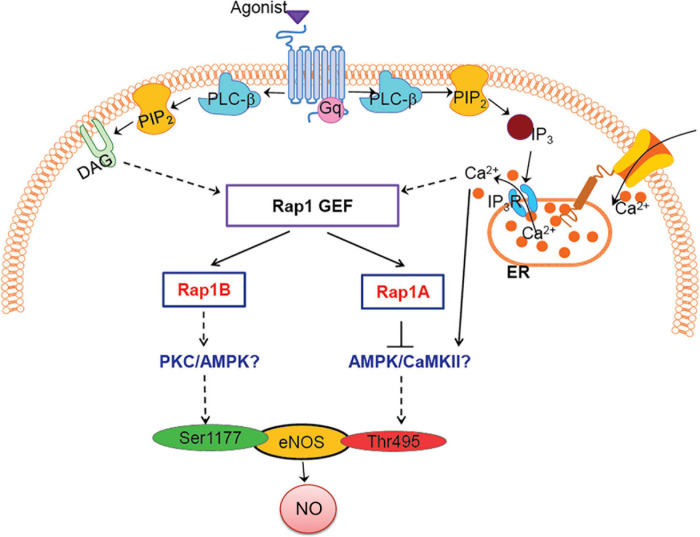
Model: how Rap1A, Rap1B contribute to NO release, role in agonist-induced eNOS activation. Two Rap1 isoforms regulate eNOS activity via distinct signaling pathways; Rap1B promotes eNOS, activating Ser1177 phosphorylation, while Rap1A suppresses Thr495 eNOS phosphorylation, preventing eNOS inhibition. Candidate kinases regulated by Rap1 isoforms, PKC/AMPK/CaMKII, are indicated in blue font.

### Rap1A Restricts eNOS Inhibition but Is Not Required for Normal Vasorelaxation

Our previous studies of Rap1 isoforms involvement in NO regulation have focused on Rap1B, the predominant Rap1 isoform in ECs (Lakshmikanthan et al., 2014, 2015). In our *ex vivo* studies, we demonstrated a partial vasorelaxation defect in EC-Rap1B KO (Lakshmikanthan et al., 2014) vs. almost complete inhibition of vasorelaxation upon deletion of both Rap1 isoforms (Lakshmikanthan et al., 2015). While this result implicated loss of Rap1A as a contributing factor to decreased NO release, the contribution of Rap1A isoform to NO release has not been directly addressed. To assess the role of Rap1A in NO release, we examined acetylcholine-induced vasorelaxation of aortic rings from Rap1A^i^**Δ**^EC^ mice. Surprisingly, we found that, unlike EC deletion of Rap1B, EC deletion of Rap1A did not lead to impaired vasorelaxation of aortic segments *ex vivo*, compared to controls (Figure 3A). EC-independent vasorelaxation to SNP was also unaffected by EC-Rap1A deficiency (Figure 3B). To further explore the relationship between Rap1A and NO release regulation, we examined the effect of Rap1A deletion on agonist-induced regulatory eNOS phosphorylation in hCAECs. We found a significant increase in eNOS-Ser1177 activating phosphorylation in siRap1A hCAECs, compared to siControls (Figures 3C,D). Interestingly, Rap1A deletion also increased inhibitory phosphorylation of eNOS-pThr495 (Figures 3E,F). In contrast, deletion of Rap1B did not significantly alter eNOS-pThr495 (Figures 3G,H). These findings demonstrate that Rap1A and Rap1B direct distinct pathways converging at eNOS activation; while Rap1B promotes the stimulatory eNOS-Ser1177 phosphorylation, Rap1A restricts the inhibitory eNOS-Thr495 phosphorylation (Figure 4).

## Discussion

The seminal findings of the study are as follows: both Rap1 isoforms contribute to NO release and thus are significant regulators of endothelial homeostasis. Moreover, the two Rap1 isoforms act *via* distinct pathways. Rap1B directly contributes to eNOS activation and NO release, acting in a signaling pathway promoting eNOS activating phosphorylation. In contrast, Rap1A restricts regulatory eNOS phosphorylation events and, in particular, restricts negative eNOS regulation. Significantly, disrupted Rap1A signaling does not functionally affect endothelial function in the presence of Rap1B—NO-dependent vasorelaxation is normal in Rap1A^iΔEC^ mice (Figure 3A). The importance of Rap1A’s modulatory function only becomes evident in the absence of Rap1B (Rap1^iΔEC^ mice), where it further impairs vasorelaxation (Lakshmikanthan et al., 2014, 2015) compared to EC-Rap1B-deficient mice (Lakshmikanthan et al., 2014). These findings suggest distinct signaling functions, converging on NO release, by the two—highly homologous—Rap1 proteins.

### eNOS Is a Target of Both Rap1 Isoforms

The compound effect of endothelial loss of both Rap1A and Rap1B isoforms is severe endothelial dysfunction and hypertension (Lakshmikanthan et al., 2015). Here, we demonstrate that this phenotype is ameliorated by restoring NO bioavailability (Figure 1). Our study demonstrates that the synergistic function of Rap1A and Rap1B occurs *via* distinct signaling pathways, converging at eNOS phosphorylation, and suggests that the two isoforms have distinct effectors. In particular, signaling leading to eNOS phosphorylation is differentially regulated by the two Rap1 isoforms. In response to GPCR agonist stimulation, similarly to EC activation by shear stress (Lakshmikanthan et al., 2015), Rap1B is rapidly activated and promotes phosphorylation on Ser1177, and its deficiency leads to decreased eNOS phosphorylation at Ser1177 and blocks NO release from ECs. In contrast, deletion of Rap1A has a dissimilar effect on eNOS phosphorylation: it increases not only activating Ser1177 phosphorylation but also inhibitory Thr495 phosphorylation.

Dual phosphorylation of Ser1177 and Thr495 determines the activity of eNOS in agonist-stimulated ECs (Fleming et al., 2001; Förstermann and Sessa, 2012). Most GPCR agonists, such as histamine, ATP, thrombin, and bradykinin, regulate phosphorylation of eNOS at Ser1177 and Thr495 *via* different protein kinases; Ser1177 phosphorylation is mediated by protein kinase C (PKC) (Gonçalves Da Silva et al., 2009) and AMP-activated protein kinase (AMPK) (Thors et al., 2004). Thr495 phosphorylation, in turn, is catalyzed by calmodulin-dependent kinase II (CaMKII) (Fleming et al., 2001; Murthy et al., 2017), and dephosphorylation is catalyzed by PP1 in a calcium-dependent manner (Fleming et al., 2001). In addition, dysregulation of the Thr495 phosphorylation site may contribute to increased superoxide, rather than NO, generation by activated eNOS (Lin et al., 2003). Increased phosphorylation of Thr495 in siRap1A ECs suggests that CaMKII activity increases in the absence of Rap1A and that Rap1A acts to suppress CaMKII. Conversely, decreased Ser1177 phosphorylation in response to agonist stimulation in siRap1B ECs implicates Rap1B in positive regulation of PKC or AMPK (Figure 4).

### Physiological Functions of Rap1 Isoforms

Our study addressed, for the first time, the role of Rap1A, the less predominant Rap1 isoform, in contribution to NO release and endothelial function. Previous studies have implicated Rap1A in the regulation of endothelial barrier and vascular integrity (Birukova et al., 2015; Lakshmikanthan et al., 2018) and FGF-dependent angiogenesis (Yan et al., 2008). However, the involvement of Rap1A in regulation of endothelial function has not been addressed—until now. Mechanistically, our study implicates Rap1A in restraining EC response to agonists that stimulate eNOS. However, the impact of Rap1A deficiency on the endothelial phenotype is largely influenced by the presence of Rap1B, as combined deficiency of both isoforms has a more extreme deleterious effect on NO release (Lakshmikanthan et al., 2015) than Rap1B deficiency alone (Lakshmikanthan et al., 2014).

Hypertension in Rap1^iΔ^ ^EC^ mice (Figure 1C; Lakshmikanthan et al., 2015) is a strong indication of the physiological significance of endothelial Rap1 to the regulation of vascular reactivity. Although most of the mechanistic studies linking eNOS were performed in large vessels, it is likely that similar mechanisms operate in small resistance vessels. Vascular resistance within tissues and organs is largely controlled by small arterioles (40–150 μm) (Durand and Gutterman, 2013). In these vessels, ACh, BK, and shear stress are major regulators of vasodilation. Thus, it is possible that in addition to activated eNOS, prostaglandins and other vasoactive substances referred to as endothelial-derived hyperpolarizing factors (EDHFs), such as hydrogen peroxide (H_2_O_2_) and epoxyeicosatrienoic acids (EETs), play a role. Whether Rap1 controls the release of other vasoactive factors will be addressed in future studies. Nonetheless, decreased NO production, also resulting from genetic interference with eNOS, results in hypertension (Huang et al., 1995; Shesely et al., 1996) and our studies underscore the importance of both Rap1 isoforms in NO production.

### Mechanisms Linking Rap1 Isoforms With NO Release

Our molecular studies point to altered kinase activities resulting in differential eNOS phosphorylation in the absence of Rap1 isoforms, but the exact mechanism remains to be elucidated. In particular, Rap1 proteins may have indirect effect on kinases modulating eNOS activity. Alternative and potentially complementary mechanisms can be envisioned. Rap1A may control NO bioavailability by inhibiting reactive oxygen species (ROS) generation. We have previously shown that in choroid epithelial cells, Rap1A can inhibit NOX-dependent activation (Wang et al., 2014), and a similar mechanism in ECs may exist. Additional studies are needed to fully explain the signaling pathways controlled by Rap1A and Rap1B and their interplay, which is necessary for normal endothelial function.

In sum, our study underscores the importance of both Rap1 isoforms in the production of NO by ECs. Rap1 isoform function is not redundant, and the two isoforms act *via* distinct pathways, converging on NO bioavailability.

## Data Availability Statement

The raw data supporting the conclusions of this article will be made available by the authors, without undue reservation.

## Ethics Statement

The animal study was reviewed and approved by the Medical College of Wisconsin Institutional Animal Use and Care Committee.

## Author Contributions

RK designed and performed eNOS phosphorylation analysis, analyzed data, and edited the manuscript. BS designed and performed L-arginine supplementation experiments and blood pressure measurements, analyzed data, and reviewed the manuscript. SL designed and performed Rap1 activity assay and eNOS phosphorylation analysis and critically reviewed the manuscript. YN designed and performed vasodilation experiments and analyzed vasodilation data. JV-V designed and performed NO measurements, analyzed data, and critically revised the manuscript. DZ designed vasodilation experiments, analyzed data, and critically reviewed the manuscript. MC designed the study, analyzed data, and wrote the manuscript. All authors contributed to the article and approved the submitted version.

## Conflict of Interest

The authors declare that the research was conducted in the absence of any commercial or financial relationships that could be construed as a potential conflict of interest.
