# Corrigendum: Distinct Signaling Functions of Rap1 Isoforms in NO Release From Endothelium

**DOI:** 10.3389/fcell.2021.741935

**Published:** 2021-08-06

**Authors:** Ramoji Kosuru, Bandana Singh, Sribalaji Lakshmikanthan, Yoshinori Nishijima, Jeannette Vasquez-Vivar, David X. Zhang, Magdalena Chrzanowska

**Affiliations:** ^1^Blood Research Institute, Versiti, Milwaukee, WI, United States; ^2^Department of Medicine, Medical College of Wisconsin, Milwaukee, WI, United States; ^3^Department of Biophysics and Redox Biology Program, Medical College of Wisconsin, Milwaukee, WI, United States; ^4^Cardiovascular Center, Medical College of Wisconsin, Milwaukee, WI, United States; ^5^Department of Pharmacology and Toxicology, Medical College of Wisconsin, Milwaukee, WI, United States

**Keywords:** endothelial (dys)function, vasodilation, nitric oxide signaling, small GTP protein, Rap1A, Rap1B

In the original article, we neglected to include the funder Director's Fellowship Award at the Blood Research Institute of Versiti, Wisconsin to Ramoji Kosuru.

The authors apologize for this error and state that this does not change the scientific conclusions of the article in any way. The original article has been updated.

## Publisher's Note

All claims expressed in this article are solely those of the authors and do not necessarily represent those of their affiliated organizations, or those of the publisher, the editors and the reviewers. Any product that may be evaluated in this article, or claim that may be made by its manufacturer, is not guaranteed or endorsed by the publisher.

